# Discovery of two new Cystobasidiaceae species (Cystobasidiales, Cystobasidiomycetes) on phylloplane from western China

**DOI:** 10.3897/mycokeys.131.185583

**Published:** 2026-04-29

**Authors:** Peng Wang, Bing-Yan Song, Chun-Yue Chai, Qiu-Hong Niu, Feng-Li Hui

**Affiliations:** 1 School of Life Science, Nanyang Normal University, Nanyang 473061, China School of Life Science, Nanyang Normal University Nanyang China; 2 Research Center of Henan Provincial Agricultural Biomass Resource Engineering and Technology, Nanyang Normal University, Nanyang 473061, China Research Center of Henan Provincial Agricultural Biomass Resource Engineering and Technology, Nanyang Normal University Nanyang China

**Keywords:** Basidiomycetous yeast, phylogenetic analysis, plant leaf, taxonomy

## Abstract

The fungal family Cystobasidiaceae, which includes the genera *Begerowomyces*, *Cystobasidium*, *Cystastrum*, *Halobasidium*, *Robertozyma*, and *Queiroziella*, comprises species that are predominantly associated with plants in tropical to cold regions. However, members of the genera *Queiroziella* and *Robertozyma* remain poorly studied. In the present study, fungal species were identified using a polyphasic approach combining phenotypic characterization and molecular phylogenetic analyses based on the D1/D2 domain of the large subunit (LSU) rRNA gene, the internal transcribed spacer (ITS) region, and the second largest subunit of RNA polymerase II (*RPB2*) gene. As a result, two novel species, *Queiroziella pini***sp. nov**. (holotype CICC 33638^T^) and *Robertozyma xinjiangensis***sp. nov**. (holotype GDMCC 2.525^T^), are proposed and described herein. Detailed descriptions, illustrations, and phylogenetic analyses of the novel taxa are provided, and comparisons with closely related species are presented. This study expands the known diversity of the family Cystobasidiaceae in China and provides a basis for future taxonomic and ecological investigations on this family.

## Introduction

Cystobasidiaceae is an ecologically important family within the class Cystobasidiomycetes, comprising more than 40 species that are most likely dimorphic, with the majority known only from their yeast morphs (Schoutteten et al. 2024). The family previously included the genera *Cystobasidium* and *Occultifur*, as well as several anamorphic *Rhodotorula* species belonging to the minuta clade (Bauer et al. 2006). Subsequently, species of the minuta clade were transferred to the genus *Cystobasidium* based on molecular phylogenetic evidence (Yurkov et al. 2015; Wang et al. 2015). More recently, molecular analyses of Cystobasidiomycetes showed that the genus *Occultifur* was polyphyletic, and that the type species of the genus, *O. internus*, belonged to the family Microsporomycetaceae. The remaining six species were reclassified into the newly established genus *Cystastrum* (Cystobasidiomycetes) (Schoutteten et al. 2024). Currently, six genera are accepted in Cystobasidiaceae, including *Begerowomyces*, *Cystobasidium*, *Cystastrum*, *Halobasidium*, *Robertozyma*, and *Queiroziella*, five of which were described relatively recently (Crous et al. 2018; Guo et al. 2019; Li et al. 2020; Schoutteten et al. 2024). Members of Cystobasidiaceae have been isolated from diverse habitats, including cold and Arctic environments, plant-associated niches, soil, fermented food, seawater, and dung-inhabiting habitats (Laich et al. 2013; Yurkov et al. 2015; Chang et al. 2019; Fotedar et al. 2019; Guo et al. 2019; Hwang et al. 2025). Filamentous morphs in Cystobasidiaceae are characterized by transversally septate basidia, presence of probasidia, tremelloid haustorial cells producing nanometer fusion pores and septal pores that are occluded by cystosomes (Sampaio et al. 1999; Bauer et al. 2006; Wang et al. 2015). Yeast morphs reproduce predominantly by polar budding and typically form pastel red to orange colonies that produce bioactive compounds like carotenoids, torulene, and torularhodine with antioxidant properties (Iurkov et al. 2008; Yurkov et al. 2008; Hwang et al. 2025). In addition, members of this family do not form basidiomata, lack fermentative ability, assimilate a wide range of carbon sources, and are unable to utilize nitrate (Yurkov et al. 2015; Hwang et al. 2025).

The genus *Queiroziella*, established by Crous et al. in 2018, currently comprises a single species, *Queiroziella brasiliensis*, which was isolated from leaves of *Portea leptantha* and *Tillandsia geminiflora* in Brazil. Phylogenetic analysis based on the D1/D2 domain of the LSU rRNA gene and the ITS region placed the genus within Cystobasidiomycetes, showing close relationships to *Sakaguchia*, *Cystobasidium*, and *Occultifur* (Crous et al. 2018). However, subsequent multi-gene phylogenetic analyses demonstrated that *Queiroziella* is more closely related to *Robertozyma*, a recently described genus within Cystobasidiaceae (Schoutteten et al. 2024). Similarly, the genus *Robertozyma* is currently represented by a single species, *Robertozyma ningxiaensis*, isolated from soil in China (Li et al. 2020). Species of *Queiroziella* and *Robertozyma* appear to be rare. Although the two genera share similar morphologies, they can be distinguished by physiological characteristics; for example, *Robertozyma* species do not assimilate raffinose, melibiose, D-ribose, or erythritol, whereas species of *Queiroziella* are able to utilize these substrates (Crous et al. 2018; Li et al. 2020).

During a survey of yeast diversity in China, five yeast isolates were obtained from leaf samples collected in Guizhou Province and the Xinjiang Uygur Autonomous Region. Molecular phylogenetic analyses based on the D1/D2 domain of the LSU rRNA gene, the ITS region, and the second largest subunit of RNA polymerase II (*RPB2*) gene, together with phenotypic characterization, revealed that these isolates represent two novel species belonging to the genera *Queiroziella* and *Robertozyma*. The novel taxa are described and illustrated below.

## Materials and methods

### Sample collection and yeast isolation

Fresh leaf samples were collected from a subtropical semi-deciduous forest in Pingtang County, Guizhou Province (26°45'26"N, 106°21'31"E), and from a mountain arboreal forest in the Tianshan Mountains, Urumqi City, Xinjiang Uygur Autonomous Region (43°17'21"N, 87°40'55"E). Each sample was placed in a sterile plastic bag, kept on ice, and transported to the laboratory within 24 hours for subsequent processing. Yeast strains were isolated from leaf surfaces using the washing and dilution method described by Jiang et al. (2024). Fresh leaves were cut into small fragments with sterile scissors and transferred to sterile 10 mL centrifuge tubes containing sterile distilled water supplemented with 0.05% (v/v) Tween 80. The samples were shaken for 10 min, and the resulting suspensions were serially diluted to 10^−2^. Aliquots (200 μl) from each dilution were spread onto yeast extract–malt extract (YM) agar (0.3% yeast extract, 0.3% malt extract, 0.5% peptone, 1% glucose, and 2% agar) supplemented with chloramphenicol (200 μg ml^−1^). Plates were incubated at 20 °C until colonies developed. Colonies with distinct yeast morphologies were selected and purified by repeated streaking on YM agar. Purified strains were preserved in YM broth containing 20% (v/v) glycerol at −80 °C.

### Phenotypic studies

Morphological, physiological, and biochemical characteristics were examined following standard methods described by Kurtzman et al. (2011). Colony morphology was observed after incubation on YM agar at 20 °C for 7 days. Cellular morphology was examined in YM broth after 3 days of incubation at 20 °C using a Leica DM2500 light microscope (Leica, Wetzlar, Germany). Mycelium formation was assessed by slide culture on corn meal agar (CMA; 2% cornmeal infusion and 2% agar) at 20 °C for 2 weeks. Ballistoconidium production was tested using the inverted-plate method (do Carmo-Sousa and Phaff 1962) on CMA at 17 °C for 2 weeks, and discharged spores were examined microscopically after 3–14 days. Sexual reproduction was examined by culturing single strains or mixed strain pairs on CMA, potato dextrose agar (PDA; 20% potato infusion, 2% glucose, and 2% agar), and V8 agar (10% V8 juice and 2% agar) at 17 °C for up to 6 weeks, with observations at 2-week intervals (Li et al. 2020). Carbon and nitrogen source assimilation tests were performed in liquid media; starved inocula were used for nitrogen assimilation tests. Glucose fermentation was assessed using Durham fermentation tubes. Growth at different temperatures was evaluated on YM agar incubated at temperatures ranging from 15 to 37 °C. Newly proposed taxa were registered in the MycoBank database (https://www.mycobank.org; accessed 26 December 2025).

### DNA extraction, PCR amplification, sequencing

Genomic DNA was extracted from yeast cells using the Ezup Column Yeast Genomic DNA Purification Kit (Sangon Biotech, Shanghai, China) according to the manufacturer’s instructions. The D1/D2 domain of the large subunit (LSU) rRNA gene, the internal transcribed spacer (ITS) region, and the second largest subunit of RNA polymerase II (*RPB2*) gene were amplified using primer pairs NL1/NL4 (Kurtzman and Robnett 1998), ITS1/ITS4 (White et al. 1990), and RPB2-5F/RPB2-7R (Wang et al. 2014), respectively. PCR amplifications were performed in 25 μl reaction mixtures containing 9.5 μl nuclease-free water, 12.5 μl 2× Taq PCR Master Mix with blue dye (Sangon Biotech, Shanghai, China), 1 μL genomic DNA template, and 1 μl of each primer. The LSU and ITS regions were sequenced following Chai et al. (2023), while *RPB2* sequences were generated as described by Wang et al. (2014). PCR products were purified using a SanPrep Column PCR Product Purification Kit (Sangon Biotech, Shanghai, China) and sequenced bidirectionally by Sangon Biotech Co., Ltd. (Shanghai, China). Forward and reverse sequences were assembled and edited using BioEdit v.7.1.3.0 (Hall 1999). All newly generated sequences were deposited in GenBank (https://www.ncbi.nlm.nih.gov/genbank/).

### Phylogenetic analyses

Sequences generated in this study were compared with reference sequences retrieved from GenBank using BLASTn searches. Reference taxa were selected based on BLAST results and recent phylogenetic studies (Crous et al. 2018; Li et al. 2020; Schoutteten et al. 2024) (Table 1). Sequences for each locus were aligned separately using MAFFT v.7 (Katoh et al. 2019) with default parameters and manually adjusted in BioEdit v.7.1.3.0 where necessary. The individual alignments were concatenated using PhyloSuite v.1.2.3 (Zhang et al. 2020).

**Table 1.** Sequences used in phylogenetic analysis. Entries in bold are newly generated in this study.

Species	Strain/clone no.	Locality	GenBank accession no.			References
			ITS	LSU	*RPB2*	
*Bannoa hahajimensis*	JCM 10336^T^	Japan	AB035897	NG_042311	KJ708146	Hamamoto et al. 2002; Wang et al. (2015)
*Bannoa syzygii*	JCM 10337^T^	Japan	AB035720	NG_058700	KJ708338	Hamamoto et al. 2002; Wang et al. (2015)
*Begerowomyces foliicola*	CGMCC 2.3164^T^	China	NR_174036	NR_174036	MK849294	Li et al. 2020
*Begerowomyces aurantius*	JCM 33898^T^	Japan	LC597190	LC597189	—	Guo et al. 2023
*Cyrenella elegans*	CBS 274.82^T^	USA	NR_145383	NG_058875	KJ708168	Wang et al. (2015)
*Cyrenella lichenicola*	KBP Y-6526^T^	Russia	MN128420	MN128420	OU562598	Kachalkin et al. 2024
*Cystobasidium alpinum*	CBS 14809^T^	Italy	NR_159815	NG_064346	—	Turchetti et al. (2018)
*Cystobasidium benthicum*	CBS 9124^T^	Japan	NR_171726	NG_059003	KJ708214	Nagahama et al. (2003); Wang et al. (2015)
*Cystobasidium calyptogenae*	JCM 10899^T^	Japan	AB025996	AB025996	KJ708218	Nagahama et al. (2003); Wang et al. (2015)
*Cystobasidium fimetarium*	DB 1489^T^	Germany	LM644067	LM644067	KJ708246	Bauer et al. (2006); Wang et al. (2015)
*Cystobasidium halotolerans*	MUCL 057192^T^	Qatar	NR_185555	MH846252	—	Fotedar et al. (2019)
*Cystobasidium iriomotense*	JCM 24594^T^	Nansei-shoto	AB726571	AB726571	—	Tanimura et al. (2018)
*Cystobasidium keelungensis*	CBS 6949^T^	Taiwan, China	FJ515193	FJ5152481	—	Chang et al. (2019)
*Cystobasidium laryngis*	CBS 2221^T^	Norway	NR_154833	KY107432	KJ708240	Nagahama et al. (2003); Wang et al. (2015)
*Cystobasidium lysiniphilum*	JCM 5951^T^	Japan	AB078501	AB078501	KJ708243	Nagahama et al. (2003); Wang et al. (2015)
*Cystobasidium minutum*	CBS 319^T^	Japan	NR_149346	NG_059005	KJ708246	Fell et al. (2000); Wang et al. (2015)
*Cystobasidium ongulense*	JCM 31527^T^	Antarctica	LC155915	LC155915	—	Tsuji et al. (2016)
*Cystobasidium pallidum*	CBS 320^T^	—	KY103146	NG_059006	KJ708253	Fell et al. (2000); Wang et al. (2015)
*Cystobasidium pinicola*	CBS 9130^T^	China	NR_154834	NG_066178	KJ708257	Zhao et al. (2002); Wang et al. (2015)
*Cystobasidium raffinophilum*	CGMCC 2.3822^T^	China	MK050389	MK050389	MK849329	Li et al. (2020)
*Cystobasidium slooffiae*	CBS 5706^T^	Hungary	NR_103568	NG_059008	KJ708266	Scorzetti et al. (2002); Wang et al. (2015)
*Cystobasidium terricola*	CGMCC 2.3823^T^	China	MK050390	MK050390	MK849330	Li et al. (2020)
*Cystobasidium tubakii*	JCM 31526^T^	Antarctica	LC155913	LC155913	—	Tsuji et al. (2016)
*Cystobasidium* sp.	CGMCC 2.3824	China	MK050391	MK050391	MK849331	Li et al. (2020)
*Cystastrum brasiliensis*	CBS 12687^T^	Brazil	KC698874	KC698874	—	Gomes et al. 2015
*Cystastrum externus*	JCM 10725^T^	Portugal	AF444567	AF1899106	KJ708199	Sampaio et al. 1999; Wang et al. (2015)
*Cystastrum kilbournensis*	CBS 13982^T^	USA	KP413162	KP413160	—	Kurtzman and Robnett 2015
*Cystastrum mephitis*	CBS 14611^T^	Slovenia	NR_158838	NG_064451	—	Šibanc et al. 2018
*Cystastrum plantarum*	CBS 14554^T^	Thailand	LC158346	LC158346	—	Khunnamwong et al. 2017
*Cystastrum tropicalis*	CBS 13389^T^	Thailand	AB921282	AB921280	—	Khunnamwong et al. 2015
*Erythrobasidium hasegawianum*	JCM 1545^T^	USA	NR_111008	AF131058	KF706534	Fell et al. (2000); Wang et al. (2015)
*Erythrobasidium yunnanense*	CBS 8906^T^	China	NR_155098	NG_059190	KJ708344	Bai et al. (2001); Wang et al. (2015)
*Halobasidium phyllophilum*	CGMCC 2.6906^T^	China	NR_200487	OP470212	OP771491	Jiang et al. 2024
*Halobasidium umbonatum*	MT 254^T^	USA	MK990658	MK990685	—	Parra (2019)
*Halobasidium xiangyangense*	GDMCC 2.231^T^	China	MH209248	MH212153	MN786640	Guo et al. (2019)
Cystobasidiomycetes sp.	BI 218	Brazil	—	EU678949	—	Direct from Genbank
‘*Cystobasidium*’ sp.	BMA 88	Brazil	—	MH908977	—	Direct from Genbank
‘*Rhodotorula*’ sp.	YM 24691	China	—	JN936880	—	Direct from Genbank
*Hasegawazyma lactosa*	CBS 5826^T^	Japan	NR_073295	NG_057668	KJ708239	Fell et al. (2000); Wang et al. (2015)
*Queiroziella brasiliensis*	CBS 14582^T^	Brazil	KY305143	KX348021	MH187958	Crous et al. 2018
***Queiroziella pini***	**NYNU 24393**	**China**	**PP849684**	**PP849683**	**PZ233425**	**This publication**
***Queiroziella pini***	**NYNU 24343** ^T^	**China**	**PP837677**	**PP849682**	**PZ233424**	**This publication**
*Queiroziella* sp.	URM 7675	Brazil	MF663139	MF663142	—	Direct from Genbank
*Queiroziella* sp.	URM 7677	Brazil	URM7677	MF663144	—	Direct from Genbank
*Queiroziella* sp.	URM 7676	Brazil	—	MF663143	—	Direct from Genbank
*Robertozyma ningxiaensis*	CGMCC 2.4451^T^	China	NR_174035	MK050392	MK849348	Li et al. 2020
***Robertozyma xinjiangensis***	**NYNU 24728^T^**	**China**	**PQ571078**	**PQ571077**	**PZ233422**	**This publication**
***Robertozyma xinjiangensis***	**NYNU 24739**	**China**	**PX712007**	**PX712008**	—	**This publication**
***Robertozyma xinjiangensis***	**NYNU 24756**	**China**	**PX712009**	**PX712010**	**PZ233423**	**This publication**
Uncultured fungus	G4n1-4	Russia	KF780606	—	—	Direct from Genbank
*Naohidea sebacea*	CBS 8477^T^	UK	NR_121324	NG_042442	KF706535	Matheny et al. (2006); Wang et al. (2015)

Strains marked with ^“T”^ are ex-type.

Phylogenetic analyses were conducted using Maximum-likelihood (ML) method based on concatenated LSU–ITS–*RPB2* dataset as well as single LSU or ITS datasets. ML analyses were performed using MEGA v.7 (Kumar et al. 2016) under the GTR+I+G substitution model. Branch support was assessed using 1,000 bootstrap replicates (Felsenstein 1985), and bootstrap values ≥ 50% were considered significant.

## Results

### Molecular phylogeny

**Figure 1.**
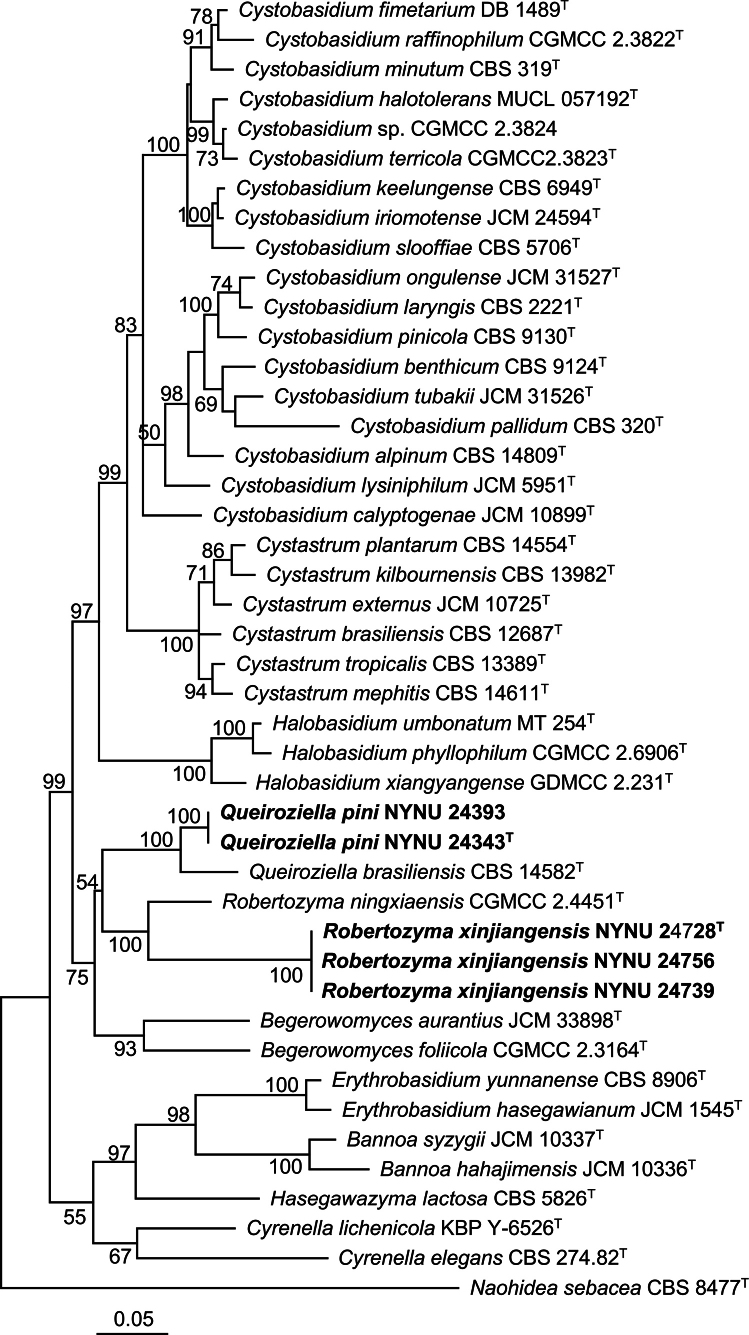
Maximum-likelihood (ML) phylogenetic tree of Cystobasidiaceae based on the concatenated LSU–ITS–*RPB2* dataset. Bootstrap support (BS) ≥ 50% are shown. *Naohidea sebacea*CBS 8477 was selected as the outgroup. New species are marked with bold. Strains marked with ^“T”^ are ex-type.

**Figure 2.**
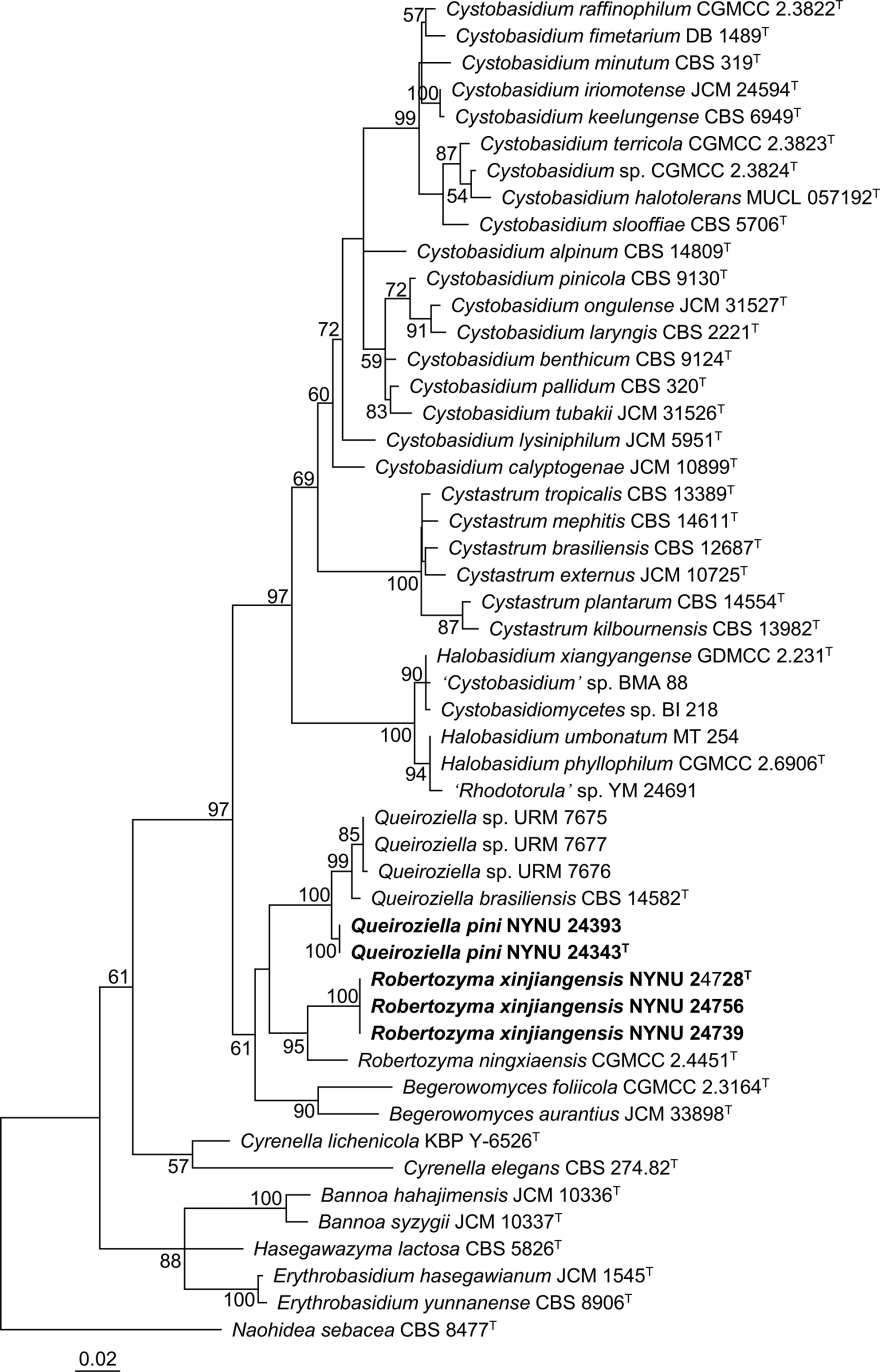
Maximum-likelihood (ML) phylogenetic tree of Cystobasidiaceae based on single LSU datasets. Bootstrap support (BS) ≥ 50% are shown. *Naohidea sebacea*CBS 8477 was selected as the outgroup. New species are marked with bold. Strains marked with ^“T”^ are ex-type.

In this study, 26 yeast strains were isolated from the collected leaf samples. Based on LSU and ITS sequence analyses, these yeast strains were assigned to eight known species—*Bullera alba*, *Derxomyces melastomatis*, *Filobasidium magnum*, *Hannaella phyllophila*, *Saitozyma flavus*, *Symmetrospora gracilis*, *Symmetrospora symmetrica*, *Tilletiopsis washingtonensis*—and two Cystobasidiaceae taxa that have not yet been formally described and, therefore, likely represent two potential new species. To determine the phylogenetic placements of two potential new species, phylogenetic analyses were conducted based on concatenated LSU–ITS–*RPB2* sequences as well as on individual LSU and ITS datasets. The concatenated alignment comprised 2160 base pairs, including 594 bp from LSU, 561 bp from ITS, and 1005 from *RPB2*. The resulting phylogenetic trees consistently indicated that the five strains represent two distinct novel species belonging to the genera *Queiroziella* and *Robertozyma*, respectively (Figs 1, 2, Suppl. material 1).

Two strains, NYNU 24393 and NYNU 24343, which shared identical LSU and ITS sequences, were placed within the genus *Queiroziella* (Figs 1, 2). These two strains clustered with *Q. brasiliensis* and three strains of a potential new species, *Queiroziella* sp. URM7675, and URM7676, in the LSU-based phylogenetic tree (Fig. 2). They differed from *Q. brasiliensis* by 7 nucleotides (nt) (~1.2%) substitutions and 17 nt (~3.2%) mismatches in the LSU and ITS regions, respectively. Comparisons with three strains of a potential new species, 10–11 nt (~1.7–1.8%) and 20 nt (~3.6–3.7%) sequence divergence were found in the LSU and ITS regions. These molecular differences support the recognition of NYNU 24393 and NYNU 24343 as a new species of *Queiroziella*, for which the name *Queiroziella pini* sp. nov. is proposed.

Three strains, NYNU 24728, NYNU 24739, and NYNU 24756, which shared identical LSU and ITS sequences, formed a separate lineage within the genus *Robertozyma* (Figs 1, 2). These strains clustered with an uncultured fungal clone G4n1-4 (KF780606) in the ITS-based phylogenetic tree (Suppl. material 1), but differed from it by six nucleotides (~1.1%) in the ITS region. The taxonomic position of clone G4n1-4 needs to be clarified in the future due to the absence of LSU sequence data at present. The difference to the known species *R. ningxiaensis* was 18 nt (~3.3%) and 29 nt (~6.3%) in the LSU and ITS regions, respectively. These levels of divergence support their recognition as a novel species of *Robertozyma*, for which the name *Robertozyma xinjiangensis* sp. nov. is proposed.

### Taxonomy

#### Queiroziella pini

Taxon classificationFungiHemipteraChlamydiaceae

C.Y. Chai & F.L. Hui sp. nov.

A88E260E-B938-5830-93FF-0E33139C420C

862001

Fig. 3

##### Etymology.

The specific epithet pini refers to *Pinus*, the plant genus from which the type strain was isolated.

**Figure 3.**
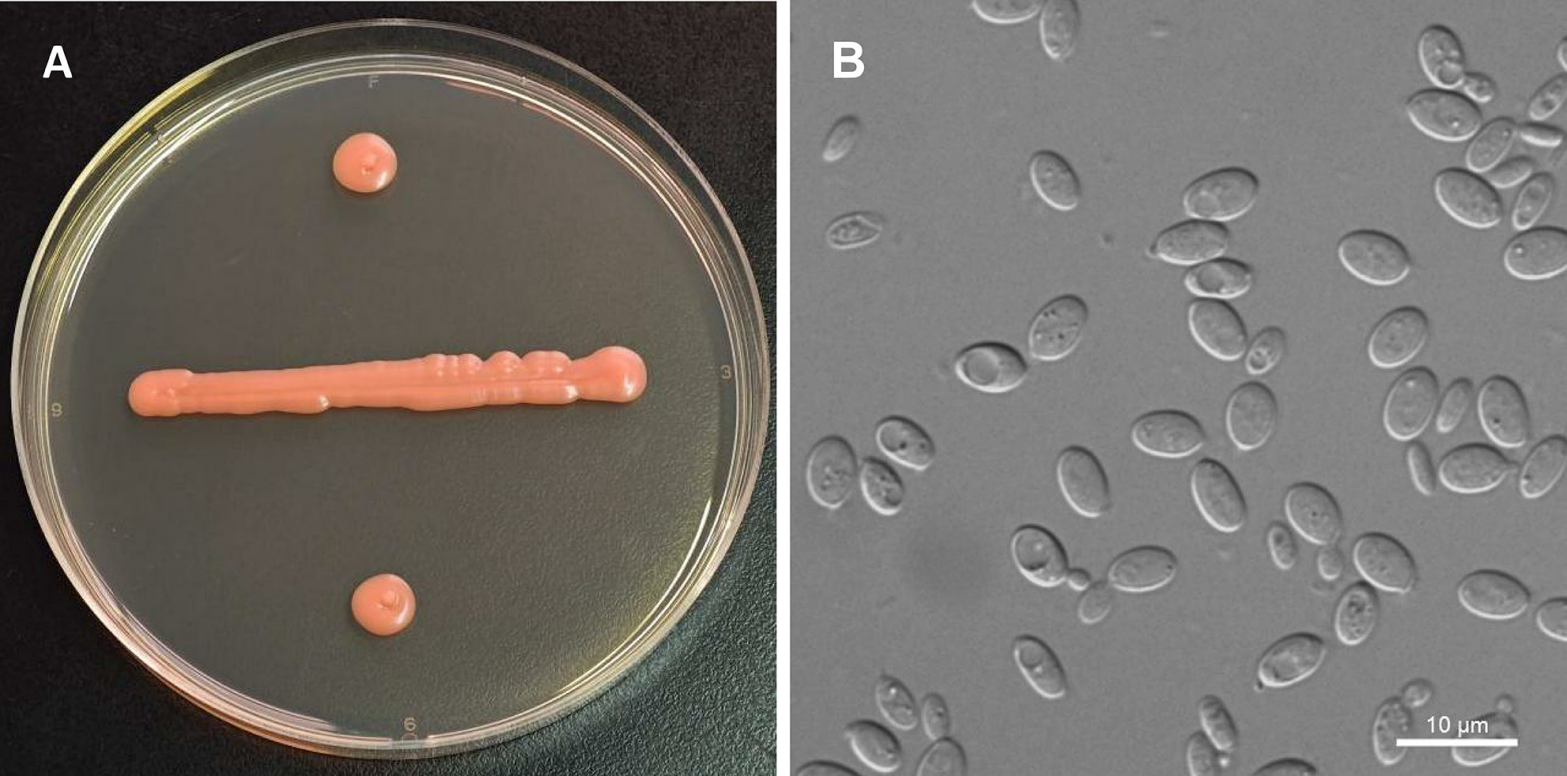
Morphology of *Queiroziella pini* (NYNU 24343). **A**. Colony on YM agar after 7 days at 20 °C; **B**. Budding cells in YM broth after 3 days at 20 °C.

##### Typus.

China • Guizhou Province, Pingtang County, Sifangjing Village, on the phylloplane of *Pinus* sp., March 2024, D. Lu, NYNU 24343 (holotype CICC 33638^T^ preserved in a metabolically inactive state, metabolically inactive ex-type culture PYCC 10055).

##### Description.

On YM agar after 7 days at 20 °C, the streak culture is pink to salmon, tough, smooth and glossy with an entire margin. In YM broth after 3 days at 20 °C, cells are ellipsoidal (3.9–8.0 × 4.2–9.4 µm) and occur singly or in pairs. Budding is polar. After a month in YM broth at 20 °C, a ring and a sediment are formed. In Dalmau plate culture on CMA at 20 °C, pseudohyphae and hyphae are not formed. Sexual structures are not observed on PDA, CMA or V8 agar at 17 °C. Ballistoconidia are not produced. Glucose fermentation is absent. The following carbon sources are assimilated: glucose, inulin (weak and delayed), sucrose, raffinose, melibiose (delayed), D-galactose, trehalose, maltose, melezitose, cellobiose, salicin, D-xylose, L-arabinose (weak), D-arabinose, 5-keto-D-gluconate, d-ribose, glycerol, erythritol, ribitol, galactitol, D-mannitol, D-glucitol, DL-lactate (weak), succinate, D-gluconate (delayed), N-acetyl-D-glucosamine, 2-keto-d-gluconate, D-glucuronate (delayed), and d-glucono-1,5-lactone. Lactose, methyl-α-D-glucoside, L-sorbose, L-rhamnose, methanol, ethanol, *myo*-inositol, citrate, and D-glucosamine are not assimilated. L-Lysine is assimilated as sole nitrogen sources. Nitrate, nitrite, ethylamine, and cadaverine are not assimilated. Growth is observed at 25 °C, but not at 30 °C. Growth on 50% (w/w) glucose-yeast extract agar is negative. Urease activity and diazonium blue B reaction are positive.

##### Additional strain examined.

China • Guizhou Province, Pingtang County, Sifangjing Village, on the phylloplane of *Ormosia henryi*, March 2024, D. Lu, NYNU 24393.

##### GenBank accession numbers.

Holotype CICC 33638^T^ (ITS: PP837677, LSU: PP849682, *RPB2*: PZ233424); additional strain NYNU 24393 (ITS: PP849684, LSU: PP849683, *RPB2*: PZ233425).

##### Note.

Physiologically, *Q. pini* differs from the closely related species *Q. brasiliensis* in its ability to assimilate salicin D-xylose, L-arabinose, galactitol, and succinate and its inability to assimilate L-sorbose and grow at 30 °C (Table 2).

**Table 2.** Physiological and biochemical characteristics of the new species and their closely related taxa.

Characteristic	*Q. pini*	*Q. brasiliensis* ^*^	*R. xinjiangensis*	*R. ningxiaensis* ^*^
Assimilation				
Inulin	d, w	s	d	–
Raffinose	+	+	–	–
Melibiose	d	s	–	–
Galactose	+	+	–	d, w
Raffinose	+	+	–	–
Salicin	+	–	d, w	d, w
L-Sorbose	–	+	–	–
D-Xylose	+	–	–	–
L-Arabinose	w	–	–	–
Ribitol	+	n	+	–
Galactitol	+	–	–	–
Succinate	+	–	+	+
D-Gluconate	d	v	d, w	–
Growth tests				
Growth at 25 °C	+	+	+	–
Growth at 30 °C	–	+	–	–

^*^Data for reference species were taken from Crous et al. (2018) and Li et al. (2020). +, positive; –, negative; d, delayed positive; v, variable; w, weakly positive; n, not available.

#### Robertozyma xinjiangensis

Taxon classificationFungiCystobasidiales

C.Y. Chai & F.L. Hui sp. nov.

DB6646E5-868C-50AD-9FDE-C81C46324707

862002

Fig. 4

##### Etymology.

The specific epithet xinjiangensis refers to the geographic origin of the ex-type strain, Wulumuqi City, Xinjiang Uygur Autonomous Region.

**Figure 4.**
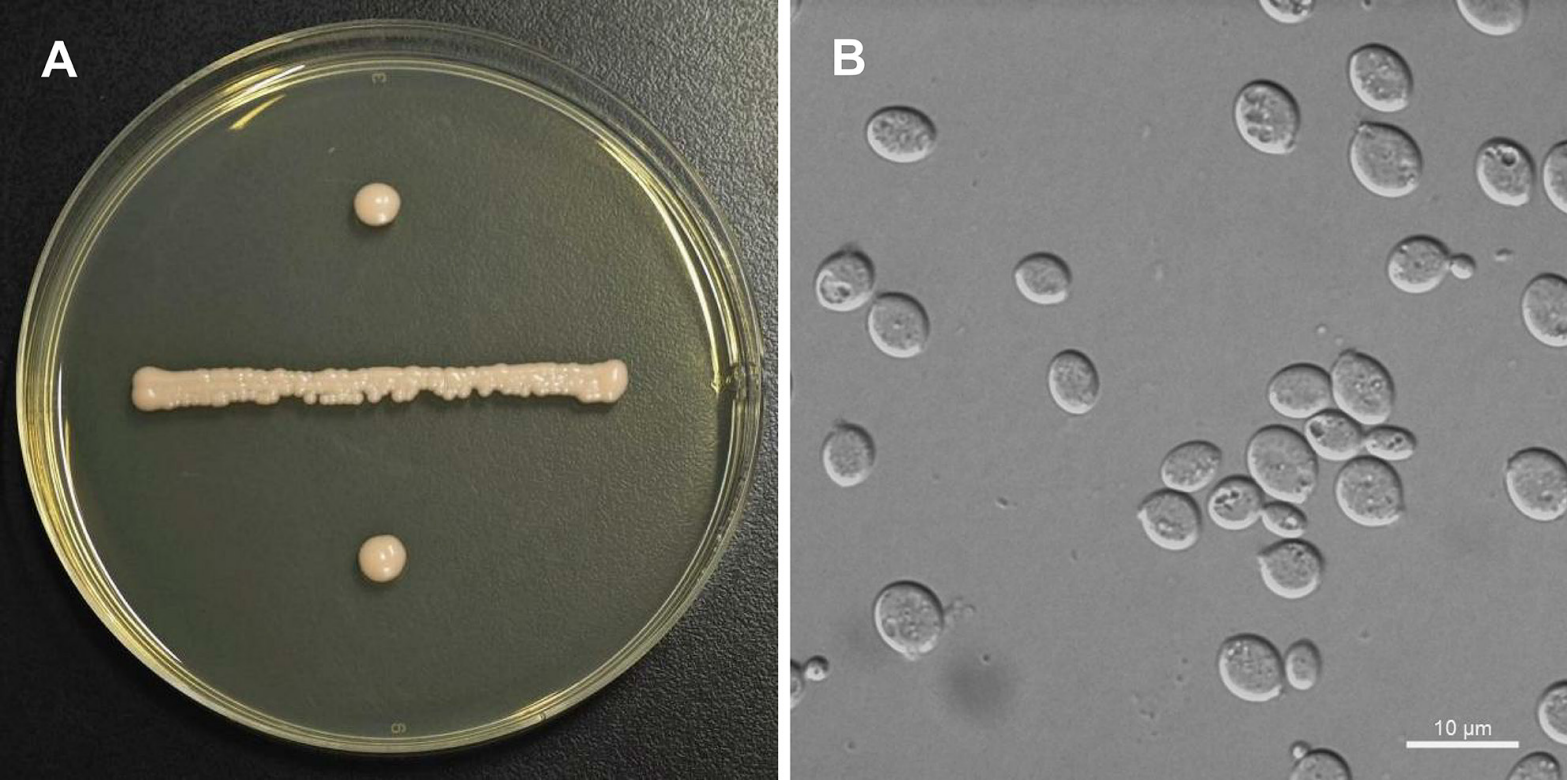
Morphology of *Robertozyma xinjiangensis* (NYNU 24728). **A**. Colony on YM agar after 7 days at 20 °C; **B**. Budding cells in YM broth after 3 days at 20 °C.

##### Typus.

China • Xinjiang Uygur Autonomous Region, Wulumuqi City, Tianshan Mountains, on the phylloplane of *Ulmus pumila*, July 2024, Z.W. Xi, NYNU 24728 (holotype GDMCC 2.525^T^ preserved in a metabolically inactive state, metabolically inactive ex-type culture PYCC 10132).

##### Description.

On YM agar after 7 days at 20 °C, streak cultures are pale yellow, smooth and butyrous, and the margin is entire. In YM broth after 3 days at 20 °C, cells are oval and ellipsoidal (4.5–5.9 × 5.9–7.8 µm) and occur singly or in pairs. Budding is polar. After a month in YM broth at 20 °C, a sediment and a ring are formed. In Dalmau plate culture on CMA at 20 °C, pseudohyphae and hyphae are absent. Sexual structures are not observed on PDA, CMA or V8 agar at 17 °C. Ballistoconidia are not produced. Glucose fermentation is absent. The following carbon sources are assimilated: glucose, inulin (delayed), trehalose, salicin (weak and delayed), D-arabinose, 5-keto-D-gluconate, glycerol, ribitol, D-mannitol, succinate, and D-gluconate (weak and delayed). Sucrose, raffinose, melibiose, D-galactose, lactose, maltose, melezitose, methyl-α-D-glucoside, cellobiose, L-sorbose, L-rhamnose, D-xylose, L-arabinose, D-ribose, methanol, ethanol, erythritol, galactitol, D-glucitol, *myo*-inositol, DL-lactate, citrate, D-glucosamine, N-acetyl-D-glucosamine, 2-keto-D-gluconate, D-glucuronate, and D-glucono-1,5-lactone are not assimilated. L-Lysine is assimilated as sole nitrogen sources. Nitrate, nitrite, ethylamine, and cadaverine are not assimilated. Growth is observed at 25 °C, but not at 30 °C. Growth on 50% (w/w) glucose-yeast extract agar is negative. Urease activity and diazonium blue B reaction are positive.

##### Additional strain examined.

China • Xinjiang Uygur Autonomous Region, Wulumuqi City, Tianshan Mountains, on the phylloplane of *Ulmus pumila*, July 2024, Z.W. Xi, NYNU 24739 and NYNU 24756.

##### GenBank accession numbers.

Holotype GDMCC 2.525^T^ (ITS: PQ571078, LSU: PQ571077, *RPB2*: PZ233422); additional strains NYNU 24739 (ITS: PX712007, LSU: PX712008) and NYNU 24756 (ITS: PX712009, LSU: PX712010, *RPB2*: PZ233423).

##### Note.

Physiologically, *R. xinjiangensis* differs from the closely related species *R. ningxiaensis* in its ability to assimilate inulin, ribitol, and D-gluconate and its inability to assimilate galactose. Furthermore, *R. xinjiangensis* is able to grow at 25 °C, whereas *R. ningxiaensis* does not exhibit growth at this temperature (Table 2).

## Discussion

In this study, five yeast strains belonging to the family Cystobasidiaceae were isolated during an investigation of phylloplane-associated fungi in western regions of China. Based on a polyphasic taxonomic approach combining phenotypic characterization and molecular phylogenetic analyses, two new species belonging to the genera *Queiroziella* and *Robertozyma* were proposed. The phylogenetic results obtained here are consistent with previous studies (Crous et al. 2018; Li et al. 2020; Schoutteten et al. 2024) and provide additional insights into the taxonomy and phylogenetic relationships within Cystobasidiaceae.

Currently, six genera have been confirmed through molecular phylogenetic analyses as members of the family Cystobasidiaceae (Schoutteten et al. 2024). The vast majority of taxa within Cystobasidiaceae are known exclusively from their yeast forms, with filamentous morphs documented only in the genera *Cystobasidium* and *Cystastrum*. Both genera are characterized by the presence of cystosomes. However, *Cystastrum* can be differentiated from the filamentous morphs of *Cystobasidium* species by the absence of thin-walled probasidia in the former genus (Li et al. 2020). The genus *Robertozyma*, along with its closely related genera, *Begerowomyces*, *Queiroziella*, and *Halobasidium*, exhibit similar morphological features. Despite this, they can be differentiated based on physiological characteristics. For instance, *Robertozyma* species do not assimilate sucrose, melezitose, D-xylose, or ethanol, whereas species of *Begerowomyces* are capable of utilizing these substrates. Moreover, *Begerowomyces* species are able to assimilate erythritol and galactitol, a trait not shared by species of *Halobasidium* and *Robertozyma*.

Previously, the genus *Queiroziella* was represented only by *Q. brasiliensis*, an epiphytic yeast isolated from leaves of *Portea leptantha*, *Tillandsia geminiflora*, and *Vriesea gigantea* in Brazil (Crous et al. 2018). The present study expands the genus by describing *Q. pini*, isolated from leaves of *Pinus* sp. and *Ormosia henryi* in a subtropical broad-leaved forest in southwestern China. In addition, three endophytic yeast strains (*Queiroziella* sp. URM7675, URM7676, and URM7677), isolated from *Handroanthus impetiginosus* in Brazil, were identified through BLAST searches against the GenBank database. These strains differ from *Q. brasiliensis* and *Q. pini* by 5–10 nucleotide differences in the LSU region and by 7–22 nucleotide differences in the ITS region, suggesting that they may represent additional potential new species within the genus *Queiroziella*. Further large-scale sampling and taxonomic studies are required to clarify the diversity, distribution, and ecological roles of this genus.

The genus *Robertozyma* was previously represented solely by *R. ningxiaensis*, isolated from soil in northwestern China (Li et al. 2020). In addition, an uncultured fungal clone (G4n1-4) from decaying wood in Russia was detected in GenBank and differs by more than six nucleotides in the ITS region from *R. ningxiaensis* and *R. xinjiangensis*, indicating the possible presence of additional *Robertozyma* species in nature. In the present study, *R. xinjiangensis* was isolated from the leaf surface of *Ulmus pumila* in the Tianshan National Forest Park, northwestern China. This distinct ecological origin suggests that *R. xinjiangensis* occupies a different ecological niche from previously known members of the genus and expands the known ecological range of *Robertozyma*. These findings highlight the importance of exploring diverse habitats for uncovering hidden yeast diversity.

## Supplementary Material

XML Treatment for Queiroziella pini

XML Treatment for Robertozyma xinjiangensis

## Additional information

### Artificial Intelligence (AI) use

The authors accept full responsibility for the content of the manuscript, including the disclosure of any use of AI.

No AI tools were used in the preparation of this manuscript.

### Author ORCIDs

P. Wang https://orcid.org/0009-0001-8289-9640

B.-Y. Song https://orcid.org/0009-0000-6802-5077

C.-Y. Chai https://orcid.org/0000-0001-7753-6223

Q.-H. Niu https://orcid.org/0000-0003-1695-7117

F.-L. Hui https://orcid.org/0000-0001-7928-3055

### Data availability

All of the data that support the findings of this study are available in the main text or Supplementary Information.

## Supplementary materials

Supplementary material 1 Maximum-likelihood (ML) phylogenetic tree of the genera *Queiroziella* and *Robertozyma* generated from the ITS sequence data Data type pdf Explanation note Bootstrap values (BS) above 50% are shown on the branches. *Begerowomyces aurantius* JCM 33898 was used as the outgroup. Type strains are denoted with the superscript “T”. Strains isolated in this study were marked in bold. Bars, 0.01 substitutions per nucleotide position. https://binary.pensoft.net/file/1613376This dataset is made available under the Open Database License (http://opendatacommons.org/licenses/odbl/1.0/). The Open Database License (ODbL) is a license agreement intended to allow users to freely share, modify, and use this Dataset while maintaining this same freedom for others, provided that the original source and author(s) are credited. Peng Wang, Bing-Yan Song, Chun-Yue Chai, Qiu-Hong Niu, Feng-Li Hui
