# Supplementary material for: Discovery of two new Cystobasidiaceae species (Cystobasidiales, Cystobasidiomycetes) on phylloplane from western China
Source: MycoKeys. 2026 Apr 29;131:287–303. doi: 10.3897/mycokeys.131.185583 (PMC13150536; doi:10.3897/mycokeys.131.185583)
Supplement: Supplementary material 1 — Maximum-likelihood (ML) phylogenetic tree of the genera Queiroziella and Robertozyma generated from the ITS sequence data [file mycokeys-131-287-s001.pdf]

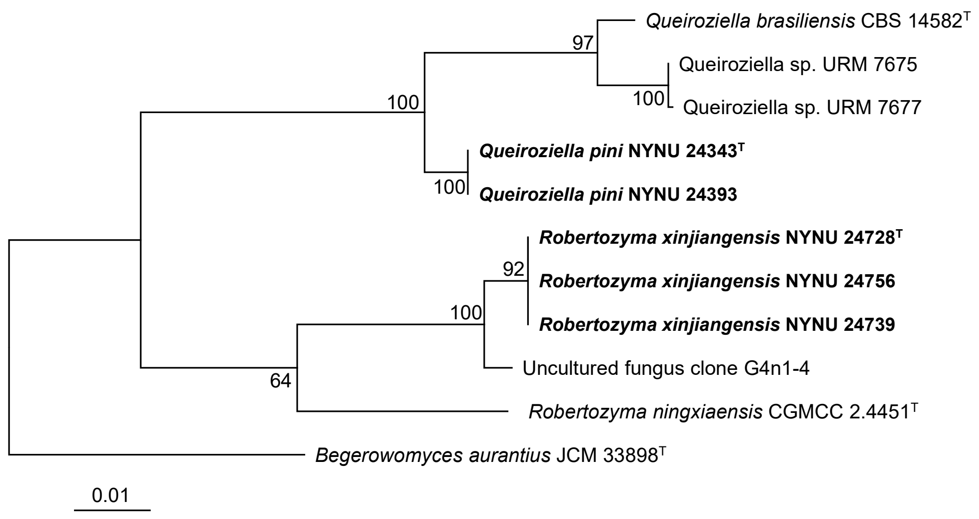

Fig. S1 Maximum-likelihood (ML) phylogenetic tree of the genera *Queiroziella* and *Robertozyma* generated from the ITS sequence data. Bootstrap values above 50% are shown on the branches. Begerowomyces aurantius JCM 33898^{T} were used as the outgroup. Type strains are denoted with the superscript “T” . Strains isolated in this study were marked in bold. Bars, 0.01 substitutions per nucleotide position.
